# Basic Analytical Modeling of Creep Strain Curves

**DOI:** 10.3390/ma16093542

**Published:** 2023-05-05

**Authors:** Rolf Sandström

**Affiliations:** Materials Science and Engineering, KTH Royal Institute of Technology, SE-10044 Stockholm, Sweden; rsand@kth.se

**Keywords:** creep strain, primary creep, tertiary creep, basic modeling

## Abstract

Creep strain versus time curves (creep curves) have traditionally been described with the help of empirical models where a number of adjustable parameters are involved. These models are simple to use, but they cannot be applied for prediction. For understanding the general behavior of primary and tertiary creep, they are still useful. In fact, the phi model can represent primary creep, and the Omega model tertiary creep for a number of materials. However, in recent years, basic analytical models have been formulated that can predict and describe creep strain data without using fitting parameters. In the paper, a review of these models is given. A number of applications of the models are also given. It is demonstrated that the basic models can quantitatively predict observations. They also provide derivations of some empirical findings.

## 1. Introduction

Creep is a slow plastic deformation that takes place even at a constant load. This is possible since, at high temperatures, dislocations can both glide and climb freely, and there is nothing that stops the deformation. There is excellent literature on creep. The textbooks by Kassner [1] and Zhang [2] can be recommended. Technically, creep is most important for steel and nickel-base alloys operating above 500 °C. Since creep is a continuous deformation process, it is of particular concern during long-term application. For fossil-fired and concentrated solar power units, the design life is up to 30 years, and for nuclear power plants up to 60 years. Since these types of plants are exposed to creep, it is critical to have accurate creep data corresponding to these long design lives. This has always been a challenge of almost prohibitive dimensions. However, with the new basic modeling of creep, it is, in fact, possible to reach these long design lives. For a review, see [3]. Some aspects of this basic modeling will be covered in the present paper.

The creep deformation is traditionally divided into three stages: primary, secondary, and tertiary. At constant load, the deformation rate (creep rate) decreases in the primary stage, it is approximately constant in the secondary stage, and it increases in the tertiary stage. To analyze mechanisms of creep deformation, the focus has been on secondary creep. The reasons are results that were formulated long ago. In two famous papers by Weertman in 1957 [4,5], it was suggested that climb and glide of dislocation would give stress exponents of 5 and 3, respectively, for the creep rate. In addition, it was already known that diffusion creep was expected to be associated with a stress exponent of 1 [6]. However, more recent results demonstrate that the climb of dislocations can show stress exponents of at least between 3 and 50, and it is still unclear if glide can be the controlling mechanism during creep [3,7]. A problem with diffusion creep is that with the low stresses involved, it is difficult to perform tests long enough to ensure that the secondary stage has been reached. New findings illustrate that dislocation creep in the primary stage can give rise to stress exponents down to 1 [8]. This demonstrates that the traditional way of identifying the main operating creep mechanism from the stress exponent in secondary creep is not very satisfactory. As will be shown in the paper, the analysis of primary creep can be quite helpful in this respect.

With modern finite element software, it is possible to design against creep with high precision. Access to creep strain versus time curves (“creep curves”) is then of great value. Important cases are when the components can allow for only small amounts of creep strain. Well-known applications are blades for gas and steam turbines where the rotor and the stator must absolutely not get in contact due to too much creep strain. Too much creep strain can also give rise to unstable structures, which must always be avoided.

Creep strain curves can have many forms, for example, for superalloys [9]. However, in most cases, they have a common form. The creep process is usually divided into three stages: primary, secondary, and tertiary. In the primary stage, the creep rate decreases with increasing time. This means that the slope of the creep versus time curve is gradually reduced with time. When the secondary stage is reached, the creep rate takes a constant value, and this also applies to the slope of the curve. For this reason, the secondary stage is also referred to as the stationary stage. In this paper, these concepts will be considered as synonyms. In the tertiary stage, the creep rate and the slope of the curve increase from the minimum value in the secondary stage. In mathematical terms, the creep curve is concave (downwards) in the primary stage, straight in the secondary stage, and convex (upwards) in the tertiary stage.

The typical form of a creep strain curve is believed to be due to the variation of the dislocation density during the creep process. For a material that is annealed and in a soft condition, the initial dislocation density is low. During creep, there is a continuous generation of new dislocations that are referred to as work hardening. In the primary stage, there is a rapid increase in the dislocation density that slows down the creep deformation. However, simultaneously with the work hardening, there is a recovery that annihilates dislocations. With increasing dislocation density, the recovery is raised faster than the work hardening. Eventually, there is a balance between work hardening and recovery. Then the secondary stage is reached, and the dislocation density becomes constant, which also gives a constant creep rate. There are many mechanisms that have been proposed to give rise to the increase of the creep rate in the tertiary stage, such as coarsening of particles and substructure and the formation of creep cavities. These mechanisms all contribute to the increase in the creep rate. However, recent results suggest another mechanism can be the main one. In the tertiary stage, the true stress in a creep specimen increases faster than the back stress from the dislocations, and this gives rise to a pronounced increase in the creep rate [10]. This will be further discussed in Section 4.

When high-temperature industrial plants exposed to creep reach their design life, there is practically always a desire to extend the operating time of the plants. To do that, the status of the creep-deformed material must be determined. This is referred to as assessing the residual life of the materials. During the creep deformation, the microstructure of the materials has changed. Practically all the changes in the microstructure reduce the creep life, and therefore these changes are referred to as creep damage. The most direct way of measuring the amount is to analyze creep curves in the tertiary stage. It is always recommended to supplement such analyses with direct observations of the microstructure to verify the findings.

The purpose of the present paper is to review basic models for creep curves that are critically needed for the long-term prediction of creep deformation. In Section 2, some empirical models for creep strain curves are presented. Both primary and tertiary stages show characteristic features. A basic model for secondary creep is summarized in Section 3. It is used in the basic models for primary and tertiary creep. The 2σ model for both primary and tertiary creep is presented in Section 4. A number of applications of the model are also given. The stress adaption model for primary creep at low stresses is derived in Section 5. The application of the model for Cu is presented in Section 6. The role of creep damage during tertiary creep is analyzed in Section 7. A creep damage model taking nucleation, growth, and linkage of cavities into account is given. It is used to reproduce the Omega model behavior during the tertiary stage in the austenitic stainless steel Sanicro 25.

## 2. Empirical Models for Creep Curves

**Figure 1:**
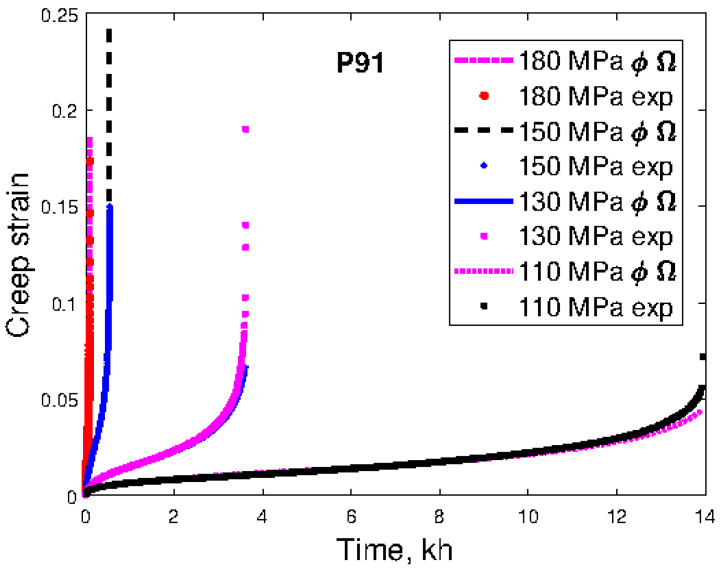
Creep strain versus time curves for the 9Cr1Mo steel P91 at 600 °C at the four stresses 110, 130, 150, and 180 MPa fitted with the ϕ and Ω models, Equations (1) and (2). Data from [11].

Creep curves show a number of characteristic features for many materials. Before modeling the creep curves, these features must be understood. Any realistic model must be able to reproduce these features. In this section, results for two materials will be analyzed. The first one is the martensitic 9Cr1Mo steel P91, which is probably the most common creep-exposed steel in modern fossil-fired power plants. The second one is the 22Cr25Ni4W1.5Co3CuNbN austenitic stainless steel Sanicro 25. It is an advanced creep-resistant steel. We will start with P91. Examples of creep curves are given in Figure 1.

**Figure 2:**
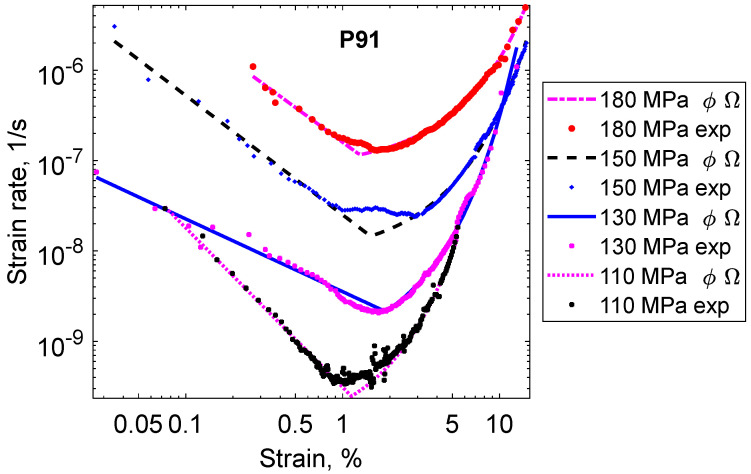
Creep rate versus strain curves for the 9Cr1Mo steel P91 at 600 °C for the same tests as in Figure 1. Double logarithmic scale.

The curves in Figure 1 show a classical behavior with primary, secondary, and tertiary stages. The primary stage is short, whereas the secondary and tertiary stages are well-developed. The creep rate is plotted versus creep strain in Figure 2.

The minimum creep rate in Figure 2 represents the secondary stage. For lower strains in the primary stage, the creep rate decreases rapidly with increasing strain. For higher strains, on the other hand, the creep rate increases in a pronounced way with increasing strain. This type of scaling obviously influences the appearance of the creep curves. In Figure 1, the secondary and the tertiary stages seem to dominate, whereas, in Figure 2, the role of the primary and tertiary stages tends to be the most important.

**Figure 3:**
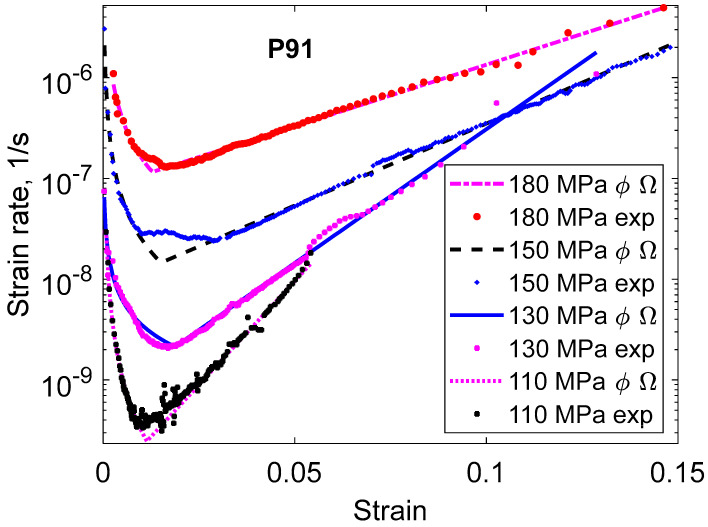
Creep rate versus strain curves for the 9Cr1Mo steel P91 at 600 °C for the same tests as in Figure 1. Semi logarithmic scale.

In the double logarithmic scale, the creep rate versus strain shows a linear behavior in the primary stage. This is a characteristic feature for a number of materials but is particularly well established for 9% and 12% Cr-steels [12,13]. It takes the following simple mathematical form.

(1) $${\overset{\cdot }{\varepsilon }}_{\mathrm{prim}}=\phi_{1}\varepsilon^{-\phi_{2}}\;\;\mathrm{phi}\;(\phi )\;\mathrm{model}$$

where ${\overset{\cdot }{\varepsilon }}_{\mathrm{prim}}$ is the creep rate in the primary stage, ε is the creep strain, and ϕ_1_ and ϕ_2_ are constants. In some form, the model was proposed many years ago. It has been generalized, and the author is now referring to it as the phi (ϕ) model [11,14]. An equally simple behavior can be found in the tertiary stage. This is illustrated in Figure 3.

In Figure 3, the tertiary stage seems to be completely dominating. In the semi-logarithmic scale, the creep rate versus strain is approximately straight in the tertiary stage. This strain dependence can be described as:

(2) $${\overset{\cdot }{\varepsilon }}_{\mathrm{tert}}=\Omega_{3}e^{\Omega_{4}\varepsilon }\;\;\mathrm{Omega}\;\mathrm{model}$$

where ${\overset{\cdot }{\varepsilon }}_{\mathrm{tert}}$ is the creep rate in the tertiary stage, ε is the creep strain, and Ω_3_ and Ω_4_ are constants. This model was proposed many years ago [15,16]. Later the model was called the Omega (Ω) model [17]. 

There are many expressions with adjustable parameters that can be used to fit creep curves. A survey can be found in [18]. However, it is recommended to use a combination of Equations (1) and (2) because then the primary and the tertiary stages can be represented separately, as well as the whole creep curves. This is shown in Figure 1, Figure 2 and Figure 3, where fitted ϕ and Ω models are included. However, if just the whole creep strain curve and nothing else should be fitted, there are many possibilities. Some examples are given in Table 1. The θ model is included since it is found in many papers in the literature. Thus, just to describe the whole creep curve, any combination of a model for primary and tertiary creep can be applied, for example, the Ω model for primary creep and the ϕ model for tertiary creep. However, such an approach is certainly not recommended.

**Figure 4:**
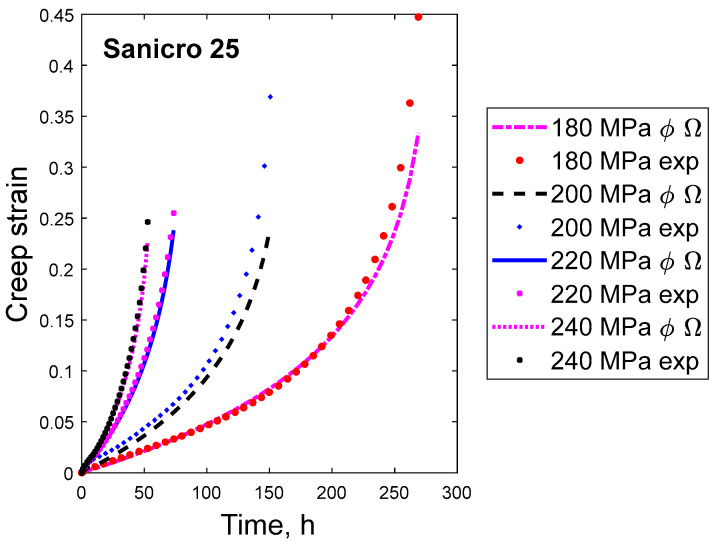
Creep strain versus time curves for the 22Cr25Ni4W1.5Co3CuNbN austenitic stainless steel Sanicro 25 at 750 °C at the four stresses 180, 200, 220, and 240 MPa fitted with the ϕ and Ω models, Equations (1) and (2). Experimental data from [24].

Examples of creep curves for Sanicro 25 are shown in Figure 4. In this case, there seems to be some secondary stage, but the diagram is dominated by tertiary creep.

**Figure 5:**
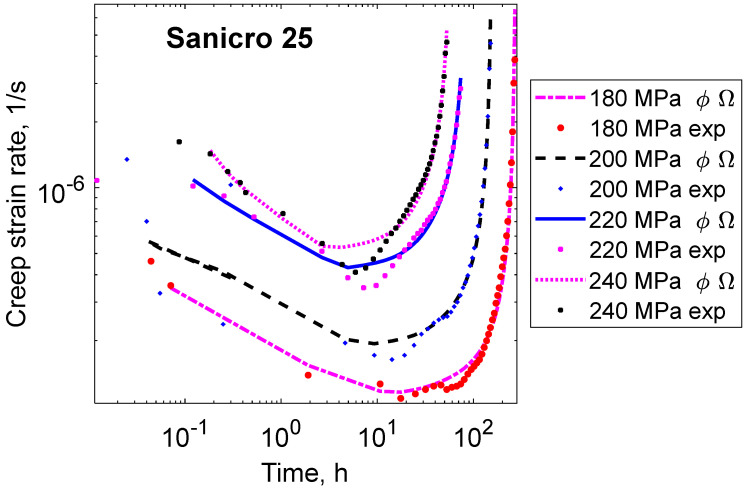
Creep rate versus time curves for the 22Cr25Ni4W1.5Co3CuNbN austenitic stainless steel Sanicro 25 at 750 °C for the same tests as in Figure 4. Double logarithmic scale.

In Figure 5, the creep rate versus time is plotted. Straight lines are also obtained in this case, where the time dependence is shown instead of the strain dependence in Figure 2. It can be seen from Table 1 that the ϕ model has the same type of exponential dependence both as a function of time and as a function of strain.

**Figure 6:**
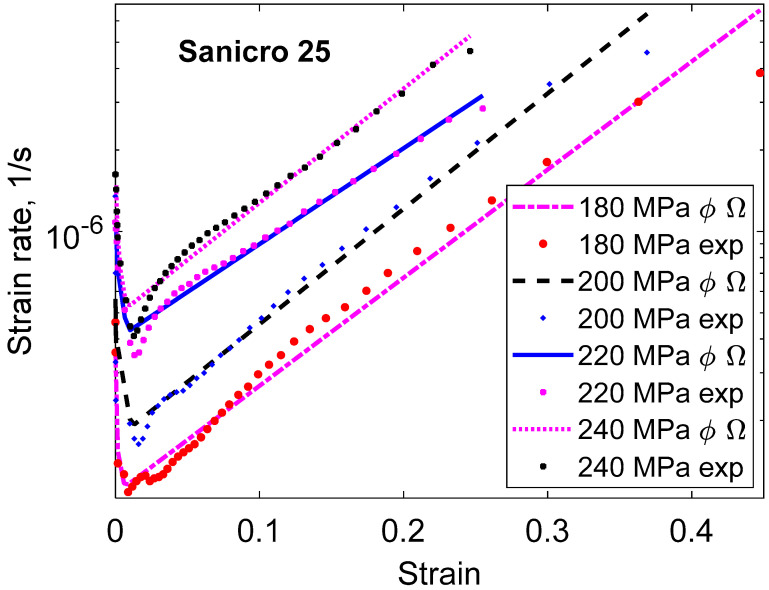
Creep rate versus strain curves for the 22Cr25Ni4W1.5Co3CuNbN austenitic stainless steel Sanicro 25 at 750 °C for the same tests as in Figure 4. Semi-logarithmic scale. Redrawn from [25] with permission from Taylor and Francis.

The behavior in the tertiary stage is given in Figure 6. It can be seen that the Ω model is closely followed also for Sanicro 25.

The ϕ model is empirical, and for a good description, the parameters have to be fitted to the experiments. However, approximate values of the parameters can be obtained in the following way:

(3) $$\;\phi_{1}={\overset{\cdot }{\varepsilon }}_{\sec}{\left(t/t_{\sec}\right)}^{-\phi_{2}}$$

where the creep rate ${\overset{\cdot }{\varepsilon }}_{\sec}$ is in the secondary stage, and *t*_sec_ is the time when the secondary stage is reached.

(4) $$\;t_{\sec}=\varepsilon_{\sec}/{\overset{\cdot }{\varepsilon }}_{\sec}$$

ε_sec_ is the strain at the start of the secondary stage. ϕ_2_ lies typically in the range of 0.75 to 0.9, and a value of 0.8 can be a starting point. If ε_sec_ and $\;{\overset{\cdot }{\varepsilon }}_{\sec}$ are known, an approximation of the parameters in the ϕ model can be obtained.

For the Ω model, a simple estimate of the parameter values can be found. First, the model in Equation (2) is rewritten in the following way:

(5) $${\overset{\cdot }{\varepsilon }}_{\mathrm{tert}}={\overset{\cdot }{\varepsilon }}_{\sec}e^{\Omega_{4}(\varepsilon -\varepsilon_{\sec})}$$

This expression takes into account that the tertiary stage starts almost at the position of the minimum creep rate. This is clearly illustrated in Figure 3 and Figure 6. This means that

(6) $$\Omega_{3}={\overset{\cdot }{\varepsilon }}_{\sec}e^{-\Omega_{4}\varepsilon_{\sec}}$$

From Table 1, it can be seen that the creep strain is a logarithmic function of the time and that the rupture can be expected to take place close to the time when the argument vanishes.

(7) $$\Omega_{4}=\frac{1}{\Omega_{3}t_{R}}=\frac{e^{\Omega_{4}\varepsilon_{\sec}}}{{\overset{\cdot }{\varepsilon }}_{\sec}t_{R}}$$

The product ${\overset{\cdot }{\varepsilon }}_{\sec}t_{R}$ can be expressed with the help of the rupture ductility factor λ_R_ and the rupture ductility ε_R_.

(8) $${\overset{\cdot }{\varepsilon }}_{\sec}t_{R}=\frac{\varepsilon_{R}}{\lambda_{R}}$$

For P91, λ_R_ is about 5 [26]. Combining Equations (7) and (8) gives

(9) $$\Omega_{4}=\frac{1}{\Omega_{3}t_{R}}=\frac{\lambda_{R}e^{\Omega_{4}\varepsilon_{\sec}}}{\varepsilon_{R}}$$

Results for fitting creep curves for 0.5Mo, 1Cr0.5Mo, and 2.25Cr1Mo gave the relation [16]

(10) $$\Omega_{4}=5.6/\varepsilon_{R}$$

which is in close agreement with Equation (9).

## 3. Basic Model for Secondary Creep

The starting point of models for primary and tertiary creep that will be discussed in the paper is the creep rate in the secondary stage. For this reason, a brief summary of a basic model for secondary creep will be given. The variation of the dislocation density ρ as a function of strain can be written as [14]

(11) $$\frac{d\rho }{d\varepsilon }=\frac{m_{T}}{bc_{L}}\rho^{1/2}-\omega \rho -2\tau_{L}M_{\mathrm{cl}}\;\rho^{2}/\overset{\cdot }{\varepsilon }$$

where ε is the strain, $\overset{\cdot }{\varepsilon }$ the creep rate, *c*_L_ a constant, ω the dynamic recovery constant, τ_L_ the dislocation line tension, and *M*_cl_ is the dislocation climb mobility. The three terms on the right-hand side of the equation represent work hardening, dynamic recovery, and static recovery. In the secondary stage, the dislocation density is constant since the conditions are stationary. To transfer the dislocation density to stresses, the Taylor equation is used.

(12) $$\sigma_{\mathrm{disl}}=\alpha m_{T}Gb\rho^{1/2}$$

where α = 0.19 is a constant [27,28], *m*_T_ the Taylor factor, *G* the shear modulus, and *b* Burgers’ vector. The dislocation stress or strength in the secondary stage σ_dislsec_ can be related to the applied stress σ.

(13) $$\sigma_{\mathrm{disl}\;\sec}=\sigma -\sigma_{{}_{i}}$$

Other strength contributions than dislocations are included in σ_i_, for example, from solid solution and precipitation hardening. Solid solution hardening is due to two effects. There is a solute drag stress since the presence of solutes reduces the velocity of climbing dislocations. In addition, there is an increase in the activation energy corresponding to the maximum interaction between solutes and dislocations. The modeling of the influence of solutes on creep is described in [3,29]. The modeling of the influence of precipitates on the creep rate is a classical issue that has found a solution that was unexpected to many people. In the past, it was assumed that there was an energy barrier that prevented dislocations from passing the precipitates. However, the final estimates of the size of this barrier were so small that they were negligible. Instead, it is the time it takes for dislocations to climb across the particles that is the critical factor. This means that only larger particles are of importance for the particle hardening since there is plenty of time for smaller particles to be passed. Details of the models for precipitation hardening during creep can be found in [3,30]. From Equations (11)–(13), the creep rate in the secondary stage can then be derived.

(14) $${\overset{\cdot }{\varepsilon }}_{\sec}=h_{\sec}(\sigma -\sigma_{i})$$

where

(15) $$h_{\sec}(\sigma )=2\tau_{L}M_{\mathrm{cl}}\frac{\sigma^{3}}{{\left(\alpha m_{T}Gb\right)}^{3}}/(\frac{m_{T}}{bc_{L}}-\omega \frac{\sigma }{\alpha m_{T}Gb})$$

The climb mobility *M*_cl_ is given as [14]

(16) $$M_{\mathrm{cl}}=\frac{D_{s}b}{k_{B}T}e^{\frac{\sigma b^{3}}{k_{B}T}}\;\exp\left(\frac{Q_{\mathrm{self}}}{R_{G}T}{\left(\frac{\sigma }{R_{\max}}\right)}^{2}\right)$$

where *D*_s_ is the self-diffusion coefficient, *k*_B_ Boltzmann’s constant, *R*_G_ is the gas constant, and *T* is the absolute temperature. *R*_max_ is the tensile strength at ambient temperatures. The last factor in Equation (16) gives strong stress dependence at high stresses. At low stresses, the temperature dependence of the creep rate is from that of the self-diffusion coefficient *D*_s_ in Equation (16). This temperature dependence is described by an Arrhenius expression in the activation energy *Q*_self_ for self-diffusion. The first two factors in Equation (16) represent the classical expression from Hirth and Lothe [31] of the mobility of vacancies and, thereby, the climb rate of dislocations.

From Equation (11), the Voce expression for a stress-strain curve can be derived, which is needed below:

(17) $$\sigma =\sigma_{i}+\sigma_{\mathrm{disl}}=\sigma_{i}+\frac{\alpha Gm_{T}{}^{2}}{\omega c_{L}}(1-\exp(-\omega \varepsilon /2))$$

where σ_i_ is the sum of other strength contributions. When deriving Equation (17), the last term in Equation (11) can be neglected. 

## 4. The 2σ Model for Primary and Tertiary Creep

To model primary and tertiary creep, it is natural to try to derive expressions from the creep rate in the secondary stage. The idea is to introduce effective stress that is combined with the expression for the stress dependence of the secondary creep rate. The choice of the effective stress σ_eff2σ_ is [3,10]

(18) $$\sigma_{\mathrm{eff}\;2\sigma }=\sigma +\sigma_{\mathrm{true}}-2\sigma_{i}-\sigma_{\mathrm{disl}}$$

where σ and σ_true_ are the nominal and true applied stress. If only primary creep is considered, σ_true_ can be taken as the nominal value σ. In the secondary stage, the effective stress is then σ–σ_i_ using Equation (13). In this way, the correct stress in the secondary stress is reproduced, cf. Equation (14). At the beginning of the primary stage, the dislocation stress is zero, and the effective stress is twice the value in the secondary stage, which usually gives quite a high creep rate. The introduction of σ_true_ in Equation (18) is necessary to handle tertiary creep. The effective stress in Equation (18) takes a high value in the initial stages of primary creep and in the later stages of tertiary creep, which is the desired behavior. Since Equation (18) is not derived directly, its accuracy must be verified by comparison to experimental results, which is done later in the section.

The relation between the true and the nominal stress is

(19) $$\sigma_{\mathrm{true}}=\sigma (1+\varepsilon )$$

When the correction for the true stress is taken into account, Equation (13) has to be modified.

(20) $$\sigma_{\mathrm{disl}\;\sec}=\sigma (1+\varepsilon_{\sec}/2)-\sigma_{i}$$

where ε_sec_ is the strain to reach the secondary stage. The dislocation stress in the secondary stage can also be obtained from Equation (17)

(21) $$\sigma_{\mathrm{disl}\;\sec}=\frac{\alpha Gm_{T}{}^{2}}{\omega c_{L}}(1-\exp(-\omega \varepsilon_{\sec}/2))$$

One must ensure that the two values for the dislocation stress in Equations (20) and (21) are the same by selecting an appropriate value for *c*_L_ in Equation (21). This implies that the expression for the dislocation stress in Equation (17) can be expressed as

(22) $$\sigma_{\mathrm{disl}}=\left[\sigma (1+\varepsilon_{\sec}/2)-\sigma_{i}\right]\frac{\left(1-\exp(-\omega \varepsilon /2)\right)}{\left(1-\exp(-\omega \varepsilon_{\sec}/2)\right)}$$

If ε = ε_sec_, Equation (20) is recovered.

In much of the creep literature, there is no clear distinction between the nominal and the true applied stress. Probably nominal stresses have been used in most cases. When the stress exponent is below, say, 7, this distinction is not so critical. However, in the power-law breakdown regime at lower temperatures, the stress exponent can reach a value of 50, and then the distinction is very important. It turns out that when trying to understand the origin of tertiary creep, the distinction is also crucial. The creep rate ${\overset{\cdot }{\varepsilon }}_{\mathrm{gen}}$ in the primary, secondary, and tertiary stages can be obtained by inserting the effective stress in Equation (18) in the expression for the secondary creep rate in Equation (15).

(23) $${\overset{\cdot }{\varepsilon }}_{\mathrm{gen}}=h_{\sec}(\sigma_{\mathrm{eff}\;2\sigma })$$

In Equation (23), the creep rate has been marked with the index “gen” to demonstrate that it is valid in all three creep stages: primary, secondary, and tertiary. 

**Figure 7:**
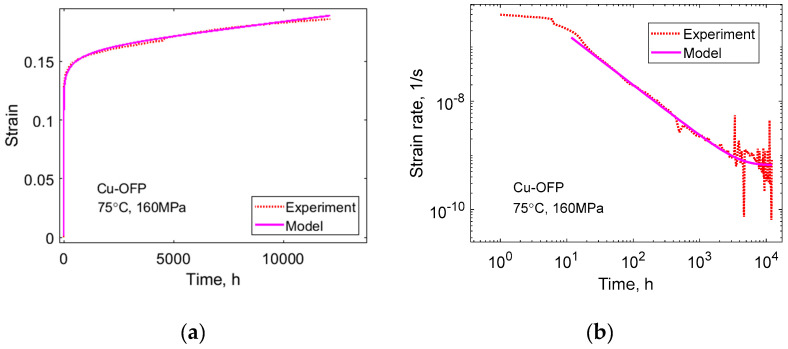
Creep test of Cu-OFP at 75 °C and 160 MPa. The creep test was interrupted after 12,000 h; (**a**) Creep strain versus time; (**b**) Creep rate versus time; Equation (23). Redrawn from [32] with permission from Elsevier.

The application of Equation (23) is illustrated in Figure 7 for Cu with 50 ppm P (Cu-OFP). The primary and the secondary stages are covered. The model gives an initial strain on loading of 0.15, in agreement with the observations in Figure 7a. There is a continuous decrease of the strain rate with increasing time until the secondary stage is reached when the curve flattens out in Figure 7b. In the primary stage, the straight curve in Figure 7b illustrates that the ϕ model is satisfied. It should be pointed out that no adjustable parameters are involved in the results from the basic models. All the parameters are defined in the models. 

In the primary stage, the dislocation stress σ_disl_ is initially low and the effective stress high, giving a high creep rate. Gradually the dislocation stress increases, and the creep rate decreases until the secondary stress is reached when there is only a slow increase. 

In the presence of a substructure with cells or subgrains, the distribution of dislocations is not fully homogenous. The reason is that dislocation on a given slip plane with opposite Burgers’ vectors or orientation moves in the opposite direction under applied stress. This means that they end up at opposite ends of the cells creating a polarized dislocation structure [10,33]. When only one type of dislocation is present, the dislocations are referred to as unbalanced or otherwise balanced. The role of unbalanced dislocations is important because they cannot meet any dislocation of the opposite type, which prevents static recovery. Balanced and unbalanced dislocation densities can essentially be derived from Equation (11), except that for unbalanced dislocation, the last term in the equation is missing [10,32]. This means that the recovery rate is slower for unbalanced dislocations, which increases the corresponding dislocation stress.

**Figure 8:**
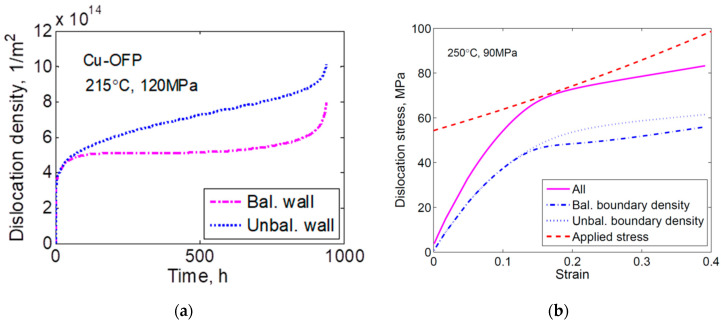
(**a**) Balanced and unbalanced dislocation densities versus time for Cu-OFP at 215 °C, 120 MPa; (**b**) Dislocation stress from balanced and unbalanced dislocation, their sum and the true applied stress minus σ_i_ as a function of strain for Cu-OFP at 250 °C, 90 MPa. Reprinted from [34] with permission of ASME.

This is illustrated in Figure 8a for creep in Cu-OFP. As can be seen, the unbalanced dislocation content increases faster than the balanced content. This means that the dislocation stress for the unbalanced content gets larger than that of the balanced content. This is demonstrated in Figure 8b.

The total contribution from both unbalanced and balanced dislocations is given as the full line in Figure 8b, which is the total dislocation stress. The dashed line is σ_true_–σ_i_. σ_i_ is essentially the yield strength which is 35 MPa. The difference between the true applied stress and dislocation stresses is the effective stress in Equation (18) up to the constant term σ–σ_i_. The difference between the dashed line and the full line is:

(24) $$\sigma_{\mathrm{true}}-\sigma_{i}-\sigma_{\mathrm{disl}}=\sigma_{\mathrm{eff}\;2\sigma }-(\sigma -\sigma_{i})$$

where Equation (18) has been rewritten. Since σ–σ_i_. is independent of strain, the difference represents the variation of the effective stress. The effective stress is minimum in the secondary stage; it is larger in the tertiary stage and, in particular, in the primary stage giving the corresponding influence on the creep rate. The increase of the effective stress in the tertiary stage is believed to be the main mechanism for the increase in the creep rate for Cu.

**Figure 9:**
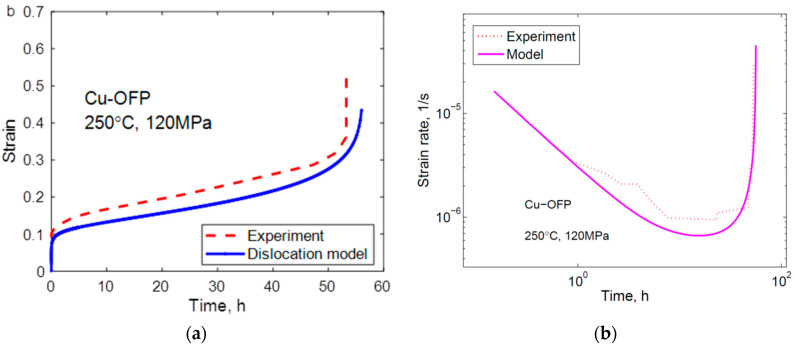
Comparison between experimental data and the model in Equation (23) for Cu-OFP at 250 °C, 120 MPa; (**a**) Creep strain versus time; (**b**) Strain rate versus creep time. Reprinted from [34] with permission of ASME.

**Figure 10:**
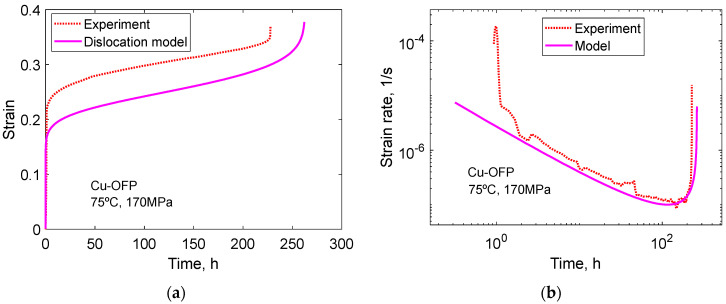
Comparison between experimental data and the model in Equation (23) for Cu-OFP at 75 °C, 170 MPa; (**a**) Creep strain versus time curve (**b**) Strain rate versus creep time. Reproduced from [35] with permission of Elsevier.

The 2σ model is compared to experimental creep curves in Figure 9 and Figure 10 for Cu-OFP at two different temperatures. Both the creep strain versus time and the creep rate versus time curves can be reproduced in an approximate way. The cusps in the experimental creep rate versus time curves are due to the necessity of reloading the creep machines after a certain strain has been reached.

## 5. Primary Creep at Low Stresses; Stress Adaption

The 2σ model seems to work well, as demonstrated in Section 4. However, the model has one limitation. The maximum value that the effective stress can take is twice the applied stress. This is no problem at high stresses where the stress exponent is high. However, at lower stresses and stress exponents, the 2σ model may not be able to cover the full observed range of creep rates in the primary stage. Fortunately, there is another model called stress adaption that is suitable for such situations. Its original form can be found in [14]. The original version involves singularities which makes it difficult to integrate. There is a new form of the model that avoids these problems. A brief derivation of the new version of the model will be given [8]. 

The starting point is the Voce Equation (17). It is rewritten in the following way:

(25) $$\sigma =\sigma_{i}+(\sigma_{\mathrm{sat}}-\sigma_{i})(1-\exp(-\omega \varepsilon /2))$$

where σ_sat_ is the maximum stress in the stress-strain curve, the saturation stress. The difference between the saturation strength and other strength contributions σ_i_ than the dislocation stress is called *K*. Both σ_i_ and *K* depend on the temperature *T* and the strain rate $\overset{\cdot }{\varepsilon }$. Both the temperature and the strain rate dependence have been found to be similar. Equation (25) is expressed in terms of a Norton expression, and the strain rate dependence is extracted.

(26) $$\sigma =\left\{\sigma_{i}(T)+K(T)(1-e^{-\omega_{m}\varepsilon /2})\right\}{\left(\frac{\overset{\cdot }{\varepsilon }}{{\overset{\cdot }{\varepsilon }}_{k}}\right)}^{1/n}$$

The temperature dependence of σ_i_ and *K* is marked. *n* is approximately the stress exponent in the Norton expansion. ${\overset{\cdot }{\varepsilon }}_{k}$ is the strain rate given at a reference stress, which is taken as the saturation stress σ_sat_ (= σ_i_ + *K*). For reasons that are explained below, the value of ω in Equation (26) can be changed, and therefore a new designation ω_m_ is introduced. A Norton expansion is also assumed for ${\overset{\cdot }{\varepsilon }}_{k}$

(27) $${\overset{\cdot }{\varepsilon }}_{k}=A_{n}{\left(\sigma_{i}(T)+K(T)\right)}^{n}$$

*A*_n_ is a constant. If Equations (26) and (27) are combined, the following expression is obtained.

(28) $$\overset{\cdot }{\varepsilon }=A_{n}{\left\{\frac{\sigma (\sigma_{i}(T)+K(T))}{\sigma_{i}(T)+K(T)(1-e^{-\omega_{m}\varepsilon /2})}\right\}}^{n}$$

This is, again, a Norton expansion that can be assumed to represent the creep rate. Now the Norton expression is replaced with the expression for the secondary creep rate, Equation (15)

(29) $${\overset{\cdot }{\varepsilon }}_{\mathrm{prim}\;\mathrm{SA}}=h_{\sec}(\frac{\sigma (\sigma_{i}(T)+K(T))}{\sigma_{i}(T)+K(T)(1-e^{-\omega_{m}\varepsilon /2})})$$

From Equation (29), the effective stress for stress adaption is found.

(30) $$\sigma_{\mathrm{eff}\;\mathrm{SA}}=\frac{\sigma (\sigma_{i}(T)+K(T))}{\sigma_{i}(T)+K(T)(1-e^{-\omega_{m}\varepsilon /2})}$$

If the strains during primary creep are sufficiently large, the value of ω_m_ can be taken as the dynamic recovery constant *ω*. However, if this is not the case, the value of ω_m_ has to be found in another way. From Equation (17), the work hardening at small strains can be obtained.

(31) $$\frac{d\sigma_{\mathrm{disl}}}{d\varepsilon }=\frac{\alpha Gm_{T}{}^{2}}{2c_{L}}$$

The dislocation stress cannot exceed the applied stress. The dislocation stress reached the applied stress at a strain that can be considered as a semi-stationary value.

(32) $$\varepsilon_{\mathrm{semi}\;\mathrm{stat}}=(\sigma -\sigma_{i})\frac{2c_{L}}{\alpha Gm_{T}{}^{2}}$$

At this stage, the exponential in Equation (30) must already be small, say 0.05. This gives the following value for ω_m_

(33) $$\omega_{m}\approx \frac{3}{\varepsilon_{\mathrm{semi}\;\mathrm{stat}}}=\frac{3\alpha Gm_{T}{}^{2}}{2c_{L}(\sigma -\sigma_{i})}$$

## 6. Application of Stress Adaption to Cu-OFP at Low Stresses

**Figure 11:**
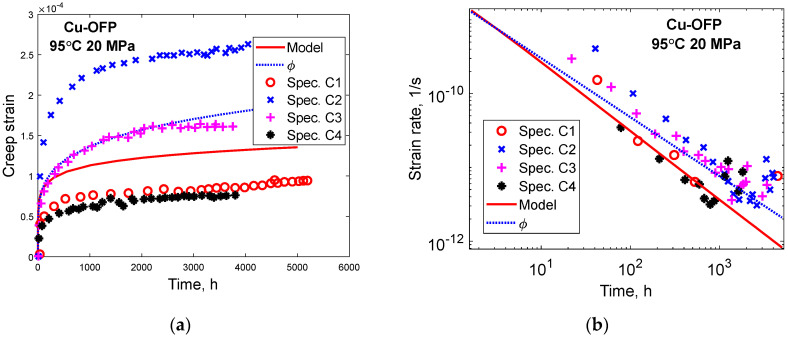
Primary creep in Cu-OFP at 95 °C, 20 MPa. Experimental data from [36]. Primary creep model in Equation (23). ϕ model according to Equation (1) with parameter values from Equations (3) and (4); (**a**) Creep strain versus time; (**b**) Creep strain rate versus time.

The creep of Cu-OFP at low stresses at 95 to 150 °C at stresses from 20 to 100 MPa was studied by Ho [36]. The creep tests were typically run for about 5000 h and then interrupted. The observed strain rates close to the time for interruption were 3 × 10^−13^ to 1.0 × 10^−10^ 1/s. As will be demonstrated below, the tests were never near stationary conditions. Creep strain versus time is shown for four specimens at 95 °C and 20 MPa in Figure 11a. Model prediction according to Equation (23) and to the ϕ model in Equation (1) is shown. In Figure 11b, the corresponding curves for the creep rate versus time are presented. Both Figure 11a,b demonstrate that the ϕ model is followed.

**Figure 12:**
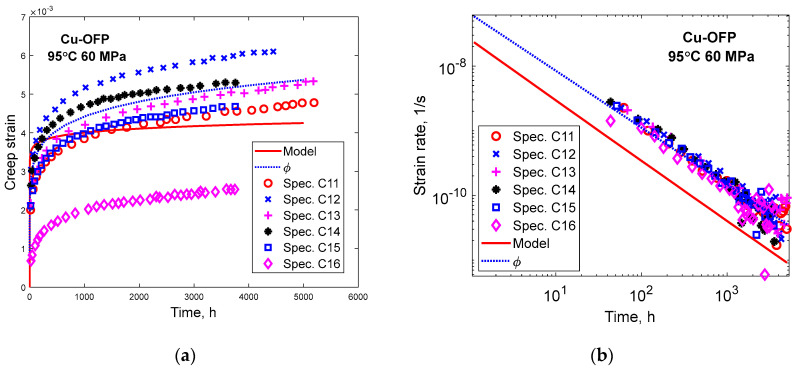
Primary creep in Cu-OFP at 95 °C, 60 MPa. Experimental data from [36]. Primary creep model in Equation (23). ϕ model according to Equation (1) with parameter values from Equations (3) and (4); (**a**) Creep strain versus time; (**b**) Creep strain rate versus time.

In Figure 12, a parallel case to Figure 11 is presented at 95 °C, 60 MPa. At these conditions, six creep tests were carried out. The results are quite similar to those in Figure 11, showing that the ϕ model is valid and that the primary creep model in Equation (23) can reproduce the creep observations reasonably well. Comparisons have also been made to the other creep tests performed by Ho [36], and similar conclusions can be drawn from them. 

With the help of Equations (14) and (15), the stationary creep rates can be estimated. They are found to be 1.3 × 10^−22^ and 1.4 × 10^−19^ 1/s corresponding to the conditions in Figure 11 and Figure 12, respectively. This is many orders of magnitude below the creep rates after 3000 to 5000 h in the Figures of 3 × 10^−12^ 1/s and 5 × 10^−11^ 1/s. As pointed out above, the creep curves in Figure 11 and Figure 12 are very far from stationary conditions. Obviously, the observations in these Figures represent data at a very early stage of primary creep. In spite of this, the data follow the ϕ model quite well, and the stress adaption model can reproduce the experiments.

## 7. Tertiary Creep with the Omega Model

There are several effects that can contribute to the increase of the creep rate in the tertiary stage. One main effect that was discussed in Section 4 is that the increase of the true stress is not fully balanced by an increase in the dislocation density. In the literature, there is much more focus on other changes in the microstructure that influence the creep rate in the tertiary stage. These changes are usually referred to as creep damage [37]. There are many mechanisms that have been proposed to contribute to the creep damage, for example, particle coarsening, increases in the subgrain size, and formation of creep cavities [38]. For the martensitic 9% Cr steels, the coarsening of fine carbonitrides (e.g., MX) has been shown to reduce the creep strength. Brittle phases such as Z-phase, Laves phase, and M_6_X carbides can be formed and decrease the creep ductility, for example, by acting as nucleation sites for cracks. In addition, the formation of brittle phases consumes alloying elements, which decreases the solid solution and precipitation hardening [39,40,41].

Quite a number of empirical or semi-empirical models to study the microstructural effects on the creep curves have been suggested. These models are almost invariably associated with a large number of adjustable parameters. This is systematized in terms of what is referred to as continuum damage mechanics. For reviews, see [42,43]. Describing the creep curves and rupture data with mathematical expressions involving a large number of parameters is usually straightforward. However, since several models based on different mechanisms can, in general, describe the experimental data fairly accurately, it gives little insight into the operating mechanisms. It is essential to use basic models without adjustable parameters to identify mechanisms. That this can be successful was illustrated for Cu in Section 4. For the important group of martensitic 9% Cr steels, creep rupture, and primary creep have been handled with basic models [44,45,46]. Unfortunately, it has not been possible to model tertiary creep in the past.

There are some intrinsic difficulties in modeling tertiary creep. Some of these difficulties will be analyzed in this section. The use of fine precipitates is the most effective way to increase the creep strength for many materials. When the materials are exposed to high temperatures, the precipitates coarsen, which reduces the creep strength. The driving force for the coarsening is the reduction of the total surface energy of the particles. The process is referred to as Ostwald ripening. For a single set of particles, its effect can be described with a simple formula:

(34) $$r_{p}^{3}=r_{0,p}^{3}+k_{p}t$$

where *r*_p_ is the average particle radius, and *r*_0,p_ is the corresponding initial value. *k*_p_ is a constant that can be precisely derived from thermodynamic properties [47]. It is proportional to the diffusion coefficient for the element that is controlling the process. 

Subgrains are also growing at high temperatures. This can be described by the following equation [48]:

(35) $$\frac{dd_{\mathrm{sub}}}{dt}=\frac{3M_{\mathrm{climb}}\tau_{L}}{2d_{\mathrm{sub}}}\cdot {\left[1-{\left(\frac{d_{\mathrm{sub}}}{d_{\mathrm{sub}\;\lim}}\right)}^{2}\right]}^{2}$$

*d*_sub_ is the subgrain diameter, and *d*_sublim_ is the limiting subgrain size due to the retarding force from particles in the sub-boundaries, which is referred to as Zener drag. The limiting subgrain size is given by [48]:

(36) $$d_{\mathrm{sub}\;\lim}=\frac{\pi r_{p}}{\gamma f_{p}}$$

*r*_p_ is the radius of particles at the subgrain boundaries, and *f*_p_ is their volume fraction. The constant γ takes a value of about 0.5 [49]. If the subgrain size is less than its limit value in Equation (36), Equation (35) is valid when the material is not exposed to stress. During creep, the subgrain size is given by a general expression relating its value to the dislocation stress.

(37) $$d_{\mathrm{sub}}=\frac{K_{\mathrm{sub}}Gb}{\sigma_{\mathrm{disl}}}$$

*K*_sub_ is a dimensionless constant with values in the interval 2 to 20 for many alloys [50]. *G* is the shear modulus, and *b* is Burgers’ vector. However, *d*_sub_ is also controlled by the upper bound in Equation (36). By increasing the volume fraction of particles in the sub-boundaries, the limiting subgrain size is reduced. In this way, the total dislocation density can be increased, which is of importance for the creep strength. In 9% Cr steels, there are M_23_C_6_ carbides in the sub-boundaries. These carbides are used to control the subgrain size and, thereby, also the creep strength.

In Figure 3 and Figure 6, the logarithm of the creep rate increases linearly with strain in the tertiary stage. This is the characteristic feature of the Omega model. There are two origins of this increase: the increase of the true stress and the presence of creep damage. The effect of the true stress is to give a slope corresponding to the creep stress exponent. In Figure 3, the stress exponent is 12. However, the slope of the curves corresponds to exponents of 28, 39, 65, and 95 for the applied stresses of 180, 150, 130, and 110 MPa, respectively. The stress exponent in Figure 6 is 5, whereas the slopes of the curves represent exponents of 9. Consequently, there is a considerable contribution from creep damage to the slope of the curves.

It is well established that particle and subgrain coarsening is of major importance for the creep damage of the 9% Cr steel P91 [51]. Equations (34) and (36) give a time-dependent contribution to the creep damage that can be shown not to be consistent with the strain dependence observed in Figure 3. Instead, strain-controlled creep damage must be considered. During creep and other plastic deformation, the number of vacancies increases. This raises the diffusion coefficient by a factor of *f_c_* in proportion to the increased number of vacancies [52]. It can be described with the following equation [52]:

(38) $$f_{c}=1+\frac{\Delta c}{c_{0}}=1+0.5\frac{\sqrt{2}K_{sub}^{2}\overset{\cdot }{\varepsilon }b^{2}}{D_{self}}\frac{G}{\sigma }$$

where *c*_0_ is the equilibrium concentration of vacancies, Δ*c* is the increased concentration of vacancies due to plastic deformation, *D*_self_ is the self-diffusion coefficient, $\overset{\cdot }{\varepsilon }$ the creep strain rate, and σ the applied stress. At lower temperatures, Equation (38) can give rise to very large increases in the creep rate of many orders of magnitude [3]. However, when Equation (38) is used in combination with the examples in Figure 3 and Figure 6, the effect is negligibly small. 

Another important damage mechanism is cavitation. During brittle rupture, nucleation, growth, and coalescence of creep cavities are believed to control the failure. A survey of modeling of creep cavitation can be found in [53]. The nucleation of creep cavities is assumed to be created by grain boundary sliding (GBS). The amount *u*_GBS_ of GBS is proportional to the creep strain ε

(39) $$u_{\mathrm{GBS}}=C_{s}\varepsilon =\frac{3\Phi }{2\xi }d_{g}\varepsilon$$

where *C*_s_ is a constant, *d*_g_ the grain size, Φ = 0.15 to 0.33 (the value increases with the creep stress exponent), and ξ = 1.36 are other constants. According to the double ledge model [54], cavities can be formed at particles in the grain boundaries and at subgrain-grain boundary junctions, and this is thermodynamically feasible, meaning that energy is released when a cavity is formed [55]. Assuming nucleation at both particles and subgrain corners, the number of nucleated cavities can be expressed as:

(40) $$n_{\mathrm{cav}}=\frac{0.9C_{s}}{d_{\mathrm{sub}}}(\frac{g_{\mathrm{sub}}}{d_{\mathrm{sub}}^{2}}+\frac{g_{\mathrm{part}}}{\lambda_{\mathrm{part}}{}^{2}})\varepsilon =B_{s}\varepsilon$$

where λ_part_ is the interparticle spacing in the grain boundaries. *g*_sub_ and *g*_part_ are the fractions of subgrain corners and particles where cavity nucleation takes place. Equation (40) can be considered as a definition of the constant *B*_s_. Equation (40) has been experimentally verified, for example, for austenitic stainless steels and Cu [56]. It should be noted that Equation (40) is strain and not time controlled.

The common form of the equation for the growth of cavities is time controlled.

(41) $$\frac{dR_{\mathrm{cav}}}{dt}=\frac{2\delta D_{\mathrm{GB}}\Omega_{a}}{k_{B}T}K_{f}(\sigma -\sigma_{0})\frac{1}{R_{\mathrm{cav}}{}^{2}}$$

where *R*_cav_ is the cavity radius in the grain boundary plane, *dR*_cav_/*dt* the growth rate, *D*_GB_ the grain boundary self-diffusion coefficient, δ the grain boundary width, and Ω_a_ the atomic volume, *k*_B_ Boltzmann’s constant, and *T* the absolute temperature. σ_0_ is the minimum stress for cavity growth (sintering stress), 2γ_s_ sin(α)/*R*_cav_, where γ_s_ is the surface energy of the cavity per unit area and α the cavity tip angle. *K*_f_ ≈ 0.2 is a constant that is a function of the cavitated area fraction. Recently, a new equation for the strain dependence of cavity growth has been proposed. It simply assumes that the cavity radius is equal to the amount of GBS. This is natural since once a cavity has been opened up by GBS, it can continue to grow due to continued GBS.

(42) $$R_{\mathrm{cav}}=C_{s}\varepsilon$$

It is notable that it is the same constant *C*_s_ that appears in Equation (39), which is known. It has been demonstrated in [57] that Equation (42) can reproduce observations for steels. There are other equations for the strain-dependent growth of cavities. They are often associated with two drawbacks. According to the principles of constrained growth, the cavities cannot grow faster than the creep deformation, and this is often not satisfied. This implies that the cavity growth rate can easily be overestimated for large cavities. On the other hand, for small cavities, the situation is the opposite. If the nucleation size for cavities is used, the cavity growth rate is negligible. If these drawbacks are present, quantitative predictions are not possible.

With Equations (40) and (42), strain-controlled nucleation and growth of cavities can be described. By combining Equations (40) and (42), the following expression for the cavitated area fraction *A*_cav_ can be obtained.

(43) $$A_{\mathrm{cav}}=g_{\mathrm{Ac}}(w/R)\int_{0}^{\varepsilon }\frac{dn_{\mathrm{cav}}}{d\varepsilon^{\prime }}(\varepsilon^{\prime })\pi R_{\mathrm{cav}}^{2}(\varepsilon ,\varepsilon^{\prime })d\varepsilon^{\prime }$$

Cavitation does not take place in all grain boundaries. The fraction of grain boundaries that are fully cavitated is referred to as *g*_Ac_. Cavities are typically elongated, which is taken into account with the ratio *w*/*R*, which is about 0.7. With the expression in Equations (40) and (42), Equation (43) can be integrated directly.

(44) $$A_{\mathrm{cav}}=g_{\mathrm{Ac}}(w/R)\frac{\pi }{3}B_{s}C_{s}{}^{2}\varepsilon^{3}$$

Equation (44) is valid for small strain. For larger strains, it must be considered that the cavity radius cannot be larger than the spacing between cavities λ_cav_

(45) $$R_{\mathrm{cav}}<\frac{\lambda_{\mathrm{cav}}}{2}=\frac{1}{2\sqrt{n_{\mathrm{cav}}}}$$

The strain dependence of the cavitated area fraction will be illustrated for Sanicro 25 for the case of 240 MPa in Figure 6. The data for Sanicro 25 are taken from [58], where the same tests as in [24] are used. The grain size *d*_g_ is 20 μm, and the distance between particles in the grain boundaries λ_part_ is 4 μm. The presence of cavitation is assumed to be heterogeneous in accordance with common experience. In the most cavitated grain boundaries where crack initiation will take place, it is assumed that every particle will generate a cavity, but for the sub-boundary–grain boundary junctions, only a tenth of them will form a cavity. The value of Φ is taken as 0.25 considering a stress exponent of 5. With these data, *C*_s_ = 5.5 μm and *B*_s_ = 3.6 × 10^11^ m^−2^ are obtained according to Equations (39) and (40).

**Figure 13:**
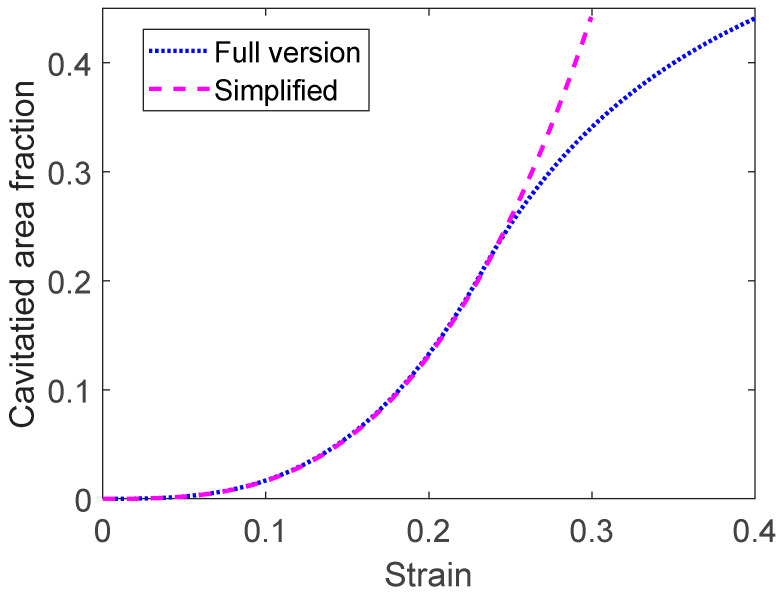
Cavitated area fraction in grain boundaries versus strain for Sanicro 25 for the case in Figure 6. The simplified expression in Equation (44) is compared to the full computed results when also the condition in Equation (45) is taken into account.

The relations for the cavitated area fraction in Equations (43) and (44) are illustrated in Figure 13 for a single boundary (*g*_Ac_ = 1). As pointed out above, Equation (44) is only valid at lower strains, in Figure 13, up to a strain of 0.25.

It is also of interest to estimate the total crack length. When the *R*_cav_ is longer than what is specified with the criterion (45), a crack is assumed to form. The total length *l*_cav_ of cavities and cracks can be estimated from the following equation:

(46) $$l_{\mathrm{cav}}=g_{\mathrm{Ac}}\int_{0}^{\varepsilon }\frac{dn_{\mathrm{cav}}}{d\varepsilon^{\prime }}(\varepsilon^{\prime })R_{\mathrm{cav}}^{}(\varepsilon ,\varepsilon^{\prime })d\varepsilon^{\prime }$$

If the condition (45) is satisfied, *l*_cav_ represents the length of cavities. If the condition is ignored, *l*_cav_ is the length of both cavities and cracks. If the condition (45) is not taken into account, Equation (46) can be integrated.

(47) $$l_{\mathrm{cav}}=g_{\mathrm{Ac}}\frac{1}{2}B_{s}C_{s}\varepsilon^{2}$$

**Figure 14:**
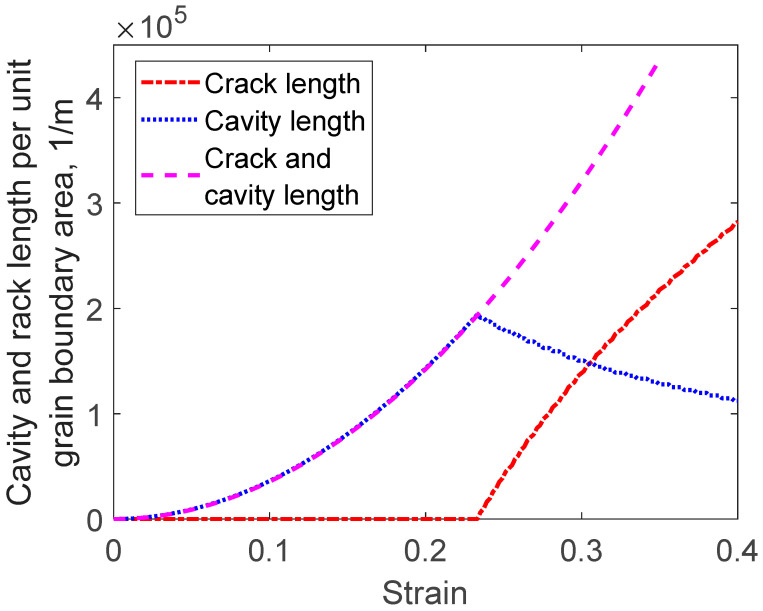
Cavity and crack length in grain boundaries versus strain for Sanicro 25 for the case in Figure 6. The expression in Equation (44) for both cavities and cracks is compared to the cavity length results when also the condition in Equation (45) is taken into account. The difference between the expressions is the crack length.

Equations (46) and (47) are exemplified in Figure 14 for a single boundary (*g*_Ac_ = 1). The length of cracks is also given. When cracks start to form by coalescence of cavities, the total length of cavities decreases. This is the reason for the drop in cavity length once cracks appear. The sum of the cavity length and the crack length is given by the expression in Equation (47).

If the cavitated area fraction is taken into account when computing the creep rate, the expression for the effective stress takes the form, cf. Equation (18)

(48) $$\sigma_{\mathrm{eff}\;2\sigma }=\sigma \frac{\left(1+\varepsilon /2\right)}{1-A_{\mathrm{cav}}}-2\sigma_{i}-\sigma_{\mathrm{disl}}$$

The dislocation stress is also influenced by *A*_cav_, cf. Equation (21)

(49) $$\sigma_{\mathrm{disl}}=\left[\sigma \frac{\left(1+\varepsilon_{\sec}/2\right)}{1-A_{\mathrm{cav}}}-\sigma_{i}\right]\frac{\left(1-\exp(-\omega \varepsilon /2)\right)}{\left(1-\exp(-\omega \varepsilon_{\sec}/2)\right)}$$

**Figure 15:**
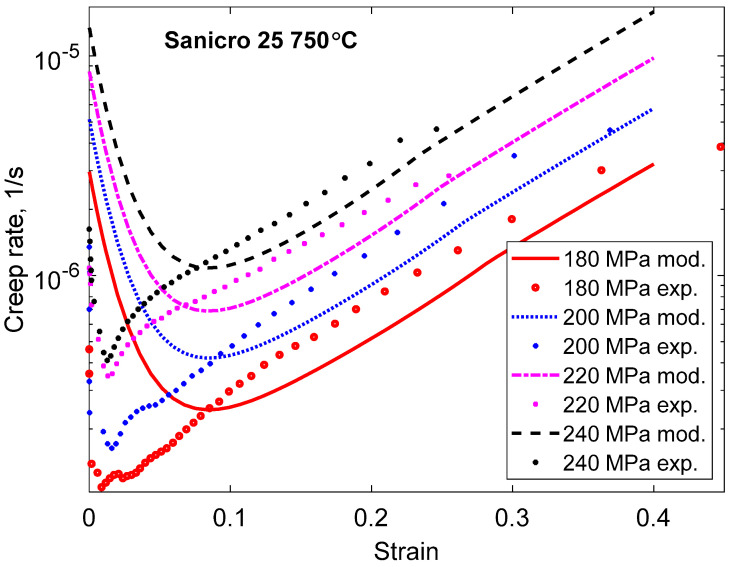
Creep rate versus strain for Sanicro 25 for the case in Figure 6 with creep tests at four stresses at 750 °C. The model in Equations (23) and (48) is compared to experimental data from [24].

The application of Equations (48) and (49) is demonstrated in Figure 15. The creep rate as a function of strain is given for the same case, as in Figure 6. The model does not reproduce the full strain dependence. In particular, the primary creep is much larger in the model than in the experiments. One reason could be that the experimental data do not include the strain on loading when the creep tests are started. It is taken into account in the model giving a strain of about 0.1 to reach the secondary stage. The general behavior of the tertiary stage is reasonably well described. In particular, the linear increase of the creep rate with increasing strain can be represented. The fraction of cavitated grain boundaries has been assumed to be 10% (*g*_Ac_ = 0.1) from data for other austenitic stainless steels [59].

## 8. Discussion 

The creep rate in the primary stage has a characteristic time dependence. In a double logarithmic diagram, it gives a linear behavior. The creep rate decreases exponentially with increasing time. This behavior is particularly well established for martensitic 9% Cr steels, as pointed out in Section 2, but it is observed for fcc alloys as well. This is referred to as the ϕ model [11]. In its integrated form, the relation has been known for a long time [19]. It was shown in Section 5 that the ϕ model is also obeyed in the very early stages of primary creep and could describe data over many orders of magnitude in time.

Two basic models for primary creep are described: the 2σ model and the stress adaption model. The 2σ model is the more general one, but it has the limitation that it cannot handle low stresses. Fortunately, the stress adaption model is suitable for low stresses. Both models have a simple structure. It is assumed that the creep rate can be computed from the stress dependence of the secondary creep rate by introducing an effective stress. For a soft material, the initial creep rate is high, and that is taken into account with a high value of the effective stress. With increasing time or strain, the effective stress is reduced until the secondary stage is reached, when it takes the value of the applied stress minus the strength contributions from other sources than the dislocations, such as solid solution and particle hardening. During the primary stage, there is a continuous increase in the dislocation density due to work hardening. The reduction of the effective stress is due to this increase in the dislocation density. For materials that are initially hard such as martensitic steels, the situation is more complex. At least two types of dislocation densities have to be introduced, free and immobile dislocations. The former type decreases with strain, but the latter type increases, making it possible to model the decreasing creep rate during the primary stage [44].

Both the 2σ model and the stress adaption model can accurately reproduce the observed decrease of the creep rate during the primary stage. The characteristic features of the ϕ model can be represented as well as the transition to the secondary stage. Even the early stages of primary creep can be described.

The 2σ model can also handle the transition to the tertiary stage and the tertiary stage itself. During the tertiary creep, there is a faster increase of the true applied stress than of the dislocation stress. In the 2σ model, this is one main reason for the increase of the creep rate in the tertiary stage.

During the tertiary stage, several changes in the microstructure take place, such as coarsening of the dislocation network, the substructure, and the precipitates. These changes are referred to as creep damage. Although there are numerous empirical models for creep damage, it is fair to say that its formation is far from well understood. There are good basic models for the changes in dislocation density, subgrain size, and radii of precipitates. However, there is a problem. The classical models for these phenomena are all time based, i.e., they are a function of time. For a number of materials, the logarithm of the creep rate is approximately linear in the creep strain. These characteristics are referred to as the Omega model. Thus, for these materials, the formation of the creep damage is strain-based, not time-based. It remains to formulate strain-based models that work at high temperatures typical for creep-exposed steels.

In the paper, a strain-based model has been formulated for one type of creep damage, namely cavitation. If cavity nucleation based on grain boundary sliding (GBS) is assumed, modeling suggests that the number of cavities formed is proportional to the creep strain. Growth of cavities is normally considered to be time-based, but there are strain-based models as well. A recent model has been applied where the cavity size is related to the amount of GBS, which is proportional to the creep strain. When the cavity length reaches the spacing distance between cavities, they are assumed to coalesce and form microcracks. In this way, a fully strain-based model for the creep damage from the cavities is obtained. It is demonstrated in the paper that if this damage model is applied to the creep model, it can reproduce the general behavior of the strain dependence of the creep rate in the tertiary stage for Sanicro 25, as shown in Figure 15. This is a major step forward, but a number of features remain to be solved, including the stress dependence of the damage in many cases.

## 9. Conclusions

Traditionally the time dependence of the creep strain and strain rate have been modeled with empirical models involving a number of adjustable parameters. These models are, in general, mathematical expressions with little or no physical background that are fitted to the experimental data. Such models are simple to use, but they have drawbacks. The results cannot be transferred from one material to another one or extrapolated. They cannot be used to predict values outside the considered set of experimental data. In the paper, basic models for the strain dependence of creep are presented that avoid these difficulties. With the basic models, quantitative predictions can be made, and a direct comparison to experimental data is possible.

- Both primary and tertiary creep show characteristic features for many materials. During the primary stage, the creep rate decreases exponentially with time, and in the tertiary stage, the logarithm of the creep rate is linear in the creep strain. These relations are referred to as the phi (ϕ) model and the Omega (Ω) model, respectively. They play a crucial role in the understanding of creep strain behavior.
- The basic 2σ model can represent primary creep and accurately describe the time dependence of the creep rate in accordance with the ϕ model. It can also handle tertiary creep, including the characteristics of the Ω model. However, it should be pointed out that the description of tertiary creep has only been verified in a limited number of cases.
- The stress adaption model is suitable to represent primary creep at low creep stresses and replaces the 2σ model in such situations. The stress adaption model also accurately reproduces the observed behavior according to the ϕ model. It is demonstrated in the paper that the stress adaption model can handle very early stages of primary creep. In this way, primary creep over many orders of magnitude in time can be described.
- The typical strain dependence in the tertiary stage, according to the Ω model, shows that the creep damage is primarily controlled by the creep strain, not by the creep time. Unfortunately, insufficient strain-dependent models are available at present. However, a strain-dependent model for cavitation is presented in the paper. The creep damage, in this case, is due to nucleation, growth, and linkage of creep cavities and, thereby, the formation of microcracks. The results for this type of damage have been inserted in the 2σ model by taking into account the role of the cavitated area fraction in the grain boundaries. In this way, it has been possible to describe the observed behavior of the creep rate in the tertiary stage for the austenitic creep-resistant steel Sanicro 25.

## Figures and Tables

**Table 1:** Empirical models for describing single creep curves [14]. Reproduced with the permission of Elsevier.

Model	Parameters	Strain Rate Versus Strain	Strain Rate Versus Time	Strain Versus Time	Refs.
ϕ model, primary	ϕ_1_, ϕ_2_	${\overset{\cdot }{\varepsilon }}_{\mathrm{prim}}=\phi_{1}\varepsilon^{-\phi_{2}}$	$\begin{array}{l} {\overset{\cdot }{\varepsilon }}_{\mathrm{prim}}=\\ \phi_{1}{\left(\phi_{1}(1+\phi_{2})t\right)}^{-\phi_{2}/(1+\phi_{2})} \end{array}$	${\left(\phi_{1}(1+\phi_{2})t\right)}^{1/(1+\phi_{2})}$	[11,19]
ϕ model, tertiary	ϕ_3_, ϕ_4_	${\overset{\cdot }{\varepsilon }}_{\mathrm{tert}}=\phi_{3}\varepsilon^{\phi_{4}}$	$\begin{array}{l} {\overset{\cdot }{\varepsilon }}_{\mathrm{tert}}=\\ \phi_{3}{\left(\phi_{3}(1-\phi_{4})t\right)}^{\phi_{4}/(1-\phi_{4})} \end{array}$	${\left(\phi_{3}(1-\phi_{4})t\right)}^{1/(1-\phi_{4})}$	[19]
Ω model, primary	Ω_1_, Ω_2_	${\overset{\cdot }{\varepsilon }}_{\mathrm{prim}}=\Omega_{1}e^{-\Omega_{2}\varepsilon }$	${\overset{\cdot }{\varepsilon }}_{\mathrm{prim}}=\frac{\Omega_{1}}{\Omega_{1}\Omega_{2}t+1}$	$\frac{\ln(\Omega_{1}\Omega_{2}t+1)}{\Omega_{2}}$	[20]
Ω model, tertiary	Ω_3_, Ω_4_	${\overset{\cdot }{\varepsilon }}_{\mathrm{tert}}=\Omega_{3}e^{\Omega_{4}\varepsilon }$	${\overset{\cdot }{\varepsilon }}_{\mathrm{tert}}=\frac{\Omega_{3}}{1-\Omega_{3}\Omega_{4}t}$	$-\frac{\ln(1-\Omega_{3}\Omega_{4}t)}{\Omega_{4}}$	[15,16,17]
θ model, primary	θ_1_, θ_2_	${\overset{\cdot }{\varepsilon }}_{\mathrm{prim}}=\theta_{2}(\theta_{1}-\varepsilon )$	${\overset{\cdot }{\varepsilon }}_{\mathrm{prim}}=\theta_{1}\theta_{2}e^{-\theta_{2}t}$	$\theta_{1}(1-e^{-\theta_{2}t})$	[21,22]
θ model, tertiary	θ_3_, θ_4_	${\overset{\cdot }{\varepsilon }}_{\mathrm{tert}}=\theta_{4}(\varepsilon +\theta_{3})$	${\overset{\cdot }{\varepsilon }}_{\mathrm{tert}}=\theta_{3}\theta_{4}e^{\theta_{4}t}$	$\theta_{3}(e^{\theta_{4}t}-1)$	[21,23]

ε is the creep strain, $\overset{\cdot }{\varepsilon }$ the strain rate, and *t* the time.

## Data Availability

Data will be made available on request.

## References

[1] Kassner M.E. (2015). Fundamentals of Creep in Metals and Alloys.

[2] Zhang J.-S. (2010). High Temperature Deformation and Fracture of Materials.

[3] Sandström R. (2018). Fundamental Models for the Creep of Metals. Creep.

[4] Weertman J. (1957). Steady-State Creep of Crystals. J. Appl. Phys..

[5] Weertman J. (1957). Steady-State Creep through Dislocation Climb. J. Appl. Phys..

[6] Herring C. (1950). Diffusional Viscosity of a Polycrystalline Solid. J. Appl. Phys..

[7] Delandar A.H., Sandström R., Korzhavyi P. (2018). The Role of Glide during Creep of Copper at Low Temperatures. Metals.

[8] Sandström R. (2023). Primary creep at low stresses in copper. Mater. Sci. Eng. A.

[9] Reed R.C. (2006). The Superalloys: Fundamentals and Applications.

[10] Sui F., Sandström R. (2018). Basic modelling of tertiary creep of copper. J. Mater. Sci..

[11] Wu R., Sandström R., Seitisleam F. (2004). Influence of Extra Coarse Grains on the Creep Properties of 9 Percent CrMoV (P91) Steel Weldment. J. Eng. Mater. Technol..

[12] Abe F., Horiuchi T., Taneike M., Sawada K. (2004). Stabilization of martensitic microstructure in advanced 9Cr steel during creep at high temperature. Mater. Sci. Eng. A.

[13] Abe F. (2009). Analysis of creep rates of tempered martensitic 9%Cr steel based on microstructure evolution. Mater. Sci. Eng. A.

[14] Sandström R. (2012). Basic model for primary and secondary creep in copper. Acta Mater..

[15] Sandstrom R., Kondyr A. (1982). Creep Deformation, Accumulation of Creep Rupture Damage and Forecasting of Residual Life for Three Mo- and CrMo-Steels. VGB Kraftw..

[16] Sandstrom R., Kondyr A. (1979). Model for tertiary-creep in Mo and CrMo-steels. Proceedings of the Conference Mechanical Behaviour of Materials, Cambridge 1979.

[17] Prager M. (1995). Development of the MPC Omega Method for Life Assessment in the Creep Range. J. Press. Vessel. Technol..

[18] Holdsworth S., Askins M., Baker A., Gariboldi E., Holmström S., Klenk A., Ringel M., Merckling G., Sandstrom R., Schwienheer M. (2008). Factors influencing creep model equation selection. Int. J. Press. Vessel. Pip..

[19] Graham A., Walles K.F.A. (1955). Relations between long and short time properties of commercial alloys. J. Iron Steel Inst..

[20] Wu R., Sandstrom R., Storesund J. (1994). Creep Strain Behavior in a 12-Percent-Crmov Steel. Mater. High Temp..

[21] Evans R.W., Wilshire B. (1985). Creep of Metals and Alloys.

[22] McVetty P.G. (1933). Factors Affecting Choice of Working Stresses for High-Temperature Service. J. Appl. Mech..

[23] McHenry D. (1943). A new aspect of creep in concrete and its application to design. Proc. ASTM.

[24] Zhang Y., Jing H., Xu L., Zhao L., Han Y., Liang J. (2017). Microstructure and texture study on an advanced heat-resistant alloy during creep. Mater. Charact..

[25] Sandström R., He J.-J. (2022). Prediction of creep ductility for austenitic stainless steels and copper. Mater. High Temp..

[26] Abe F. (2021). Influence of boron nitride inclusions and degradation in creep rupture strength on creep rupture ductility of Gr.122. Mater. High Temp..

[27] Lavrentev F. (1980). The type of dislocation interaction as the factor determining work hardening. Mater. Sci. Eng..

[28] Orlová A. (1991). On the relation between dislocation structure and internal stress measured in pure metals and single phase alloys in high temperature creep. Acta Met. Mater..

[29] Korzhavyi P., Sandström R. (2015). First-principles evaluation of the effect of alloying elements on the lattice parameter of a 23Cr25NiWCuCo austenitic stainless steel to model solid solution hardening contribution to the creep strength. Mater. Sci. Eng. A.

[30] Sui F., Sandström R. (2019). Creep strength contribution due to precipitation hardening in copper–cobalt alloys. J. Mater. Sci..

[31] Hirth J.P., Lothe J. (1967). Theory of Dislocations.

[32] Sandström R. (2016). The role of cell structure during creep of cold worked copper. Mater. Sci. Eng. A.

[33] Sandström R. (2022). Formation of Cells and Subgrains and Its Influence on Properties. Metals.

[34] Sandström R., Sui F. (2021). Modeling of Tertiary Creep in Copper at 215 and 250 °C. J. Eng. Mater. Technol..

[35] Sandström R. (2017). Formation of a dislocation back stress during creep of copper at low temperatures. Mater. Sci. Eng. A.

[36] Ho E.T.C. (2000). A Study of Creep Behaviour of Oxygen-Free Phosphorus-Doped Copper under Low-Temperture and Low-Stress Test Conditions.

[37] Cane B.J., Williams K.R., Miller K.J., Smith R.F. (1980). Creep damage accumulation and life assessment of a ½Cr½Mo¼V steel. Mechanical Behaviour of Materials.

[38] Ashby M.F., Dyson B.F., Valluri S.R., Taplin D.M.R., Rao P.R., Knott J.F., Dubey R. (1984). Creep damage mechanics and micromechanisms. Fracture 84.

[39] Vanaja J., Laha K., Mythili R., Chandravathi K.S., Saroja S., Mathew M.D. (2012). Creep deformation and rupture behaviour of 9Cr-1W-0.2V-0.06Ta Reduced Activation Ferritic-Martensitic steel. Mater. Sci. Eng. A.

[40] Aghajani A., Somsen C., Eggeler G. (2009). On the effect of long-term creep on the microstructure of a 12% chromium tempered martensite ferritic steel. Acta Mater..

[41] Abe F. (2004). Bainitic and martensitic creep-resistant steels. Curr. Opin. Solid State Mater. Sci..

[42] Meng Q., Wang Z. (2019). Creep damage models and their applications for crack growth analysis in pipes: A review. Eng. Fract. Mech..

[43] Lemaître J., Desmorat R. (2010). Engineering Damage Mechanics: Ductile, Creep, Fatigue and Brittle Failures.

[44] Magnusson H., Sandström R. (2007). Creep Strain Modeling of 9 to 12 Pct Cr Steels Based on Microstructure Evolution. Met. Mater. Trans. A.

[45] Magnusson H., Sandström R. (2007). The Role of Dislocation Climb across Particles at Creep Conditions in 9 to 12 Pct Cr Steels. Met. Mater. Trans. A.

[46] Sandstrom R., Magnusson H. (2012). Basic model for creep deformation in 12Cr1MoV steels. 12th International Conference on Creep and Fracture of Engineering Materials and Structures.

[47] Agren J., Clavaguera-Mora M.T., Golcheski J., Inden G., Kumar H., Sigli C. (2000). Application of computational thermodynamics to phase transformation nucleation and coarsening. Calphad Comput. Coupling Phase Diagr. Thermochem..

[48] Sandstrom R. (1977). Subgrain Growth Occurring by Boundary Migration. Acta Metall. Mater..

[49] Brown L.M., Ham R.K. (1971). Strengthening Methods in Crystals.

[50] Koneva N., Starenchenko V., Lychagin D., Trishkina L., Popova N., Kozlov E. (2008). Formation of dislocation cell substructure in face-centred cubic metallic solid solutions. Mater. Sci. Eng. A.

[51] Maruyama K., Chen R., Yaguchi M., Yoshimi K. (2023). A simulation of softening during creep exposure of grade 91 steel in a time range over 100,000 h around 600 °C. Int. J. Press. Vessel. Pip..

[52] Mecking H., Estrin Y. (1980). The effect of vacancy generation on plastic deformation. Scr. Met..

[53] Sandström R., He J. (2017). Survey of Creep Cavitation in fcc Metals. Study of Grain Boundary Character.

[54] Sandström R., Wu R. (2013). Influence of phosphorus on the creep ductility of copper. J. Nucl. Mater..

[55] Lim L. (1987). Cavity nucleation at high temperatures involving pile-ups of grain boundary dislocations. Acta Met..

[56] Sandström R. (2023). Cavitation during creep-fatigue loading. Mater. High Temp..

[57] Sandström R. (2022). Basic Creep-Fatigue Models Considering Cavitation. Trans. Indian Natl. Acad. Eng..

[58] Zhao L., Song K., Zhang Y., Meng S., Xu L., Han Y., Jing H. (2019). Creep Rupture Assessment of New Heat-Resistant Sanicro 25 Steel Using Different Life Prediction Approaches. J. Mater. Eng. Perform..

[59] He J., Sandström R. (2016). Creep cavity growth models for austenitic stainless steels. Mater. Sci. Eng. A.

